# Discrepancy in the suppressive function of regulatory T cells in allergic asthmatic vs. allergic rhinitis subjects upon low-dose allergen challenges

**DOI:** 10.3389/falgy.2023.1296601

**Published:** 2023-12-01

**Authors:** Martin Klein, Sophie Plante, Marie-Ève Boulay, Louis-Philippe Boulet, Jamila Chakir

**Affiliations:** Centre de Recherche de l’Institut Universitaire de Cardiologie et de Pneumologie de Québec, Université Laval, Quebec City, QC, Canada

**Keywords:** bronchoalveolar lavage fluid (BALF), Tregs, allergy, allergen exposure, Foxp3, IL-10

## Abstract

**Background:**

Regulatory T cells (Tregs) contribute to the maintenance of immunological tolerance. There is evidence of impaired function of these cells in people with asthma and allergy. In this study, we evaluated and compared the function of Tregs in allergic asthmatic and allergic non-asthmatic patients, both before and after low-dose allergen challenges.

**Methods:**

Three groups of subjects were recruited for a baseline evaluation: healthy controls without allergy or asthma, allergic asthmatic subjects, and allergic non-asthmatic subjects. All of them were subjected to expiratory flow measurements, sputum induction, and blood sampling. In addition, both groups of allergic subjects underwent low-dose allergen challenges. Tregs were isolated from whole blood using CD4^+^CD25^high^ and CD127^low^ staining. The suppression function was measured by flow cytometry. The levels of IL-10, IFN-γ, IgG4, IgA, and TGF-β were measured using ELISA, and sputum Foxp3 was evaluated using qRT-PCR.

**Results:**

The suppressive function of Tregs in healthy controls was significantly higher than in allergic asthmatic or allergic non-asthmatic subjects. Repeated exposure to low doses of allergen increased the suppressor function of Tregs in allergic non-asthmatic subjects but decreased it in allergic asthmatic subjects. Foxp3 gene expression was increased in induced sputum in allergic non-asthmatic subjects, whereas it did not change in asthmatic subjects. Serum IL-10 level was decreased in allergic asthmatic subjects after allergen challenge but not in allergic non-asthmatic subjects. IFN-γ level increased upon allergen challenge in allergic non-asthmatic subjects. IgG4 level was higher in allergic non-asthmatic subjects than in allergic asthmatic subjects.

**Conclusions:**

Low-dose allergen challenges stimulate the suppressor function of Tregs in non-asthmatic allergic subjects but not in allergic asthmatic subjects.

## Introduction

Allergic rhinitis and asthma are primarily mucosal diseases triggered by repeated aeroallergen antigen exposure in the upper and lower respiratory tracts. They generate a surge in immune response, involving innate and adaptive immunity components such as innate lymphoid cell type (ILC2), activated T helper type 2 (Th2) cells, eosinophils, IgE-producing B cell, mast cells, and basophil degranulation. These inflammatory cascades are usually balanced by an immune regulation mediated by anti-inflammatory cells maintaining allergen tolerance. In allergic diseases, allergen tolerance is disrupted because of the breakdown of usually operative immune regulatory mechanisms, which leads to aberrant and uncontrolled inflammatory responses. Regulatory T cells (Tregs) represent a specific subset of T cells that contribute to the maintenance of this tolerance. These cells can be classified into naturally occurring and inducible Tregs deriving from thymus and peripheral lymphoid tissues, respectively. Both express forkhead transcription factor (Foxp3) and CD25, but their IL-7 receptor (CD127) expression is low (1).

In pediatric asthma, regulatory T cells and Foxp3 expression are decreased in bronchoalveolar lavage fluid (BALF), and their suppressive function is impaired (2). Similar observations have been reported in several studies on the peripheral blood and sputum of asthmatic adults (3–5). In humans, blood peripheral CD4^+^CD25^+^ Treg cells derived from atopic donors (mainly allergic rhinitis) have been shown to be suppressive in allergen-stimulated cultures. This suppressive activity is similar to that in healthy controls (6). In contrast, Ling et al. have shown that Foxp3 Tregs are defective in atopic individuals. This defect is markedly visible during the pollen season in pollen-sensitive atopic subjects (7). A more recent study has shown that Foxp3 expression in the nasal fluid of allergic rhinitis patients is lower than in healthy individuals outside of pollen season (8) and increases during pollen season (9). Although increased Foxp3 expression in allergic rhinitis upon allergen exposure suggests the establishment of a counterregulatory mechanism, it is still insufficient to control the whole allergic inflammation. In contrast, Foxp3 expression in asthmatic patients remains low upon allergen exposure (10, 11), suggesting a worsened impaired function of T regulatory cells compared with that of allergic rhinitis patients. In line with this finding, the role of repeated allergen exposure on the suppressive function of Tregs remains to be explored. To evaluate the effect of repeated low-dose allergen challenges on Treg function, we exposed allergic asthmatic and allergic non-asthmatic (allergic rhinitis) subjects to low-dose allergen challenges and compared them with non-asthmatic and non-allergic subjects.

## Material and methods

### Subject characteristics

A total of 10 mild steroid-naïve allergic asthmatic subjects, 13 allergic non-asthmatic subjects, and 10 healthy control subjects were included in this study. All were non-smokers and none had a respiratory tract infection for at least 1 month prior to the study. Allergic subjects had a positive reaction to one or more allergens on prick tests and none of them were under allergen immunotherapy (AIT) before or during the study protocol. Asthmatic subjects had a history of asthma for at least 6 months, as defined by the criteria of the American Thoracic Society (ATS) (12). They were using only inhaled *β*_2_ agonists on an as-needed basis for their asthma treatment. They had a PC_20_ [the provocative concentration of methacholine inducing a 20% fall in forced expiratory volume in 1 s (FEV_1_) ≤8 mg/ml (tidal breathing method). Allergic non-asthmatic subjects had normal airway responsiveness (as shown by a PC_20_ methacholine >16 mg/ml) and they had never experienced any asthma symptoms or taken any asthma medication in the past. Informed consent was obtained from each volunteer, and the protocol was approved by the ethics committee of our institution, the IUCPQ (CER#20398). The baseline characteristics of the subjects are presented in Table 1. They participated in the allergen challenges and none of them experienced any allergic or asthmatic symptoms during the challenge days. No significant change in FEV_1_ was observed throughout the study period (less than 5% within and between visits in both groups).

**Table 1:** Subject characteristics.

Group	Age (years)	Sex (M/F)	FEV_1_ (% pred)		PC_20_ (mg/ml)	
			Baseline	Post challenge	Baseline	Post challenge
Allergic asthmatics	27.6 ± 1.6	1/9	102.4 ± 4.0	105.7 ± 4.9	3.5 ± 1.2	3.2 ± 0.9
Allergic non-asthmatics	24.7 ± 0.9	6/7	103.6 ± 4.7	104.0 ± 6.8	57.4 ± 10.4	47.4 ± 8.3
Healthy controls	35.4 ± 3.5	5/5	102.4 ± 5.1	—	63.3 ± 13.3	—

F, female; M, male.

### Study design

On a baseline visit, the subjects underwent allergy skin prick tests, spirometry, and methacholine broncho-provocation. Induced sputum and blood sampling were also obtained. We specified for each subject whether all data were obtained for each sampling and we included only those subjects for whom we had all data, as specified in Supplementary Table S1.

Both groups of allergic subjects underwent “low-dose” whole-lung allergen challenge on four consecutive days. Induced sputum was obtained on days 2 and 4, 6 h post challenge, and blood samples were obtained only 6 h following the last day of challenge (Supplementary Figure S1).

### Skin prick tests and titration

Atopy was determined from allergy tests with 26 common aeroallergens [trees, grasses, cats, house dust mites (*Dermatophagoides pteronyssinus* and *Dermatophagoides farinae*)]. Normal saline and histamine were used as negative and positive controls, respectively. The skin wheal diameter was recorded for each allergen at 10 min as the mean of two perpendicular measurements. A positive response was defined as a skin wheal diameter of 3 mm or more (Supplementary Table S1). When the prick test indicated sensitivity to several allergens, the allergen inducing the largest skin wheal diameter was chosen for the challenge protocol, and when several allergens induced the same skin wheal diameter, we chose one of them (Supplementary Table S2).

To determine the allergen dose to be given on bronchial allergen challenge, skin titration was done with the allergen to which the subject reacted the most on prick tests. Serial two-fold dilutions of the allergen were applied on the forearm, and the skin wheal diameter was measured at 15 min. The smallest concentration with a 2 mm diameter in wheal response was defined as the end-point titration and was used to determine the allergen concentration to be inhaled.

### Spirometry and methacholine inhalation test

Baseline FEV_1_ and forced vital capacity (FVC) were measured according to the ATS criteria (12). Methacholine bronchial challenge was performed as described by Juniper et al. (13). Briefly, following a 2-min inhalation of 0.9% saline, increasing concentrations of methacholine were inhaled for 2 min via a Wright nebulizer (Roxon Meditech, Montreal, QC, Canada) delivering 0.13 ml/min. FEV_1_ was measured at 30 and 90 s following inhalation or until FEV_1_ had increased. The test was stopped when a ≥20% fall in FEV_1_ from the lowest post-saline value was recorded or when the last methacholine dose was administered. The response was expressed as the PC_20_ methacholine obtained from the log dose–response curve.

### Low-dose allergen challenges

Aerosolized allergen was inhaled via a Wright nebulizer (Roxon Meditech) calibrated to deliver 0.13 ml/min. The first allergen inhalation dose was determined using the Cockcroft equation, which takes into account the end-point titration and PC_20_ (14). “Low-dose” lung allergen challenge was performed as described previously (15). Briefly, subjects visited the clinic on four consecutive mornings to undergo their allergen provocation. Three allergen doses were given for inhalation, corresponding to nine, eight, and seven doubling doses below the calculated PD_20_ (the provocative dose of allergen inducing a 20% fall in FEV_1_). The inhalation lasted for 2 min and FEV_1_ was measured 10 min following the inhalation. If FEV_1_ levels did not fall by more than 5% from the highest baseline FEV_1_, the next concentration was given; otherwise, the test was stopped. FEV_1_ was measured at 10-min intervals for 30 min following inhalation of the last allergen concentration.

### Cell isolation

Peripheral blood samples (150 ml) were obtained from all three groups of subjects at baseline and from allergic non-asthmatic and allergic asthmatic subjects after 3 days of low-dose allergen challenges. Serum was also obtained from clotted blood. Peripheral blood mononuclear cells (PBMCs) were isolated by Lymphocyte Separation Media density gradient centrifugation (Wisent). Monocytes were separated from total lymphocytes by adhesion for 1 h at 37°C. CD4^+^CD25^−^ and CD4^+^CD25^+^CD127^low^ were isolated using CD4^+^ T cell isolation Kit II and the CD4^+^CD25^+^CD127^dim/−^ Regulatory T cell Isolation Kit according to the manufacturer's instructions (Miltenyi Biotec). The purity rate of the isolated Tregs (CD4^+^CD25^+^) was >90%. The expression of CD4, CD25, CD127, and Foxp3 was analyzed by flow cytometry using anti-CD4 (GK1.5), anti-CD25 (REA570), anti-CD127 (REA614) (Miltenyi Biotec), and anti-Foxp3 (PCH101) (eBiosciences) using Coulter EPICS XL-MCL and Expo32 software (Beckman Coulter).

### CFSE labeling

Lyophilized CellTrace™ CFSE (Carboxyfluorescein diacetate succinimidyl ester) (Invitrogen) was diluted in dimethylsulfoxide (DMSO) right before use (5 mM working solution). Freshly isolated CD4^+^CD25^−^ were resuspended in PBS/0.1% BSA at 1 × 10^6^ cells/ml, and 1 μl/ml of CFSE stock solution was added for a final concentration of 5 μM. Labeling was done according to the manufacturer's protocol. Cells were washed and resuspended in RPMI 1640 (Invitrogen) for proliferation assay.

### Proliferation and suppression assay

96-well round-bottom plates (Corning Life Sciences) were coated with a 10 μg/ml anti-CD3 antibody (eBiosciences). CFSE-labeled CD4^+^CD25^−^ T cells were cultured at 2 × 10^4^ cells/well with 4 × 10^4^ irradiated autologous monocytes. Unlabeled CD4^+^CD25^+^ regulatory T cells at a ratio of 1:1 were added to the CFSE-labeled CD4^+^CD25^−^ T cells. After 5 days of culture, the supernatants were frozen and cells were harvested, washed, and analyzed by flow cytometry for obtaining CFSE signals on Coulter EPICS XL-MCL using Expo32 software (Beckman Coulter).

### Enzyme-linked immunosuppression assay

The levels of immunoglobulin A(IgA), total IgG4, IL-10, and transforming growth factor-beta (TGF-β) were measured in serum from the three groups of subjects at baseline and after allergen challenge for atopic and asthmatic subjects. IL-4, IL-10, and IFNγ levels were measured in supernatants from suppression assays using ELISA (RnD systems) following the manufacturer's directions and analyzed on a ThermoMax Microplate Reader using SoftMax Pro software (Molecular Device).

### Induced sputum and qRT-PCR

Sputum was induced and processed using the method described by Pin et al. (16) and modified by Pizzichini et al. (17) by Inhalation of hypertonic saline at baseline, and 6 h after the second and fourth days of allergen challenge. Briefly, mucus plugs were selected from saliva and treated with dithiothreitol (DTT). Total cell count and viability were determined using the trypan blue exclusion method. Cells were placed in an RLT buffer (Qiagen, Toronto, ON, Canada). Total RNA was extracted using an RNAspin extraction kit (GE) following the manufacturer's protocol. The RNA concentration of each sample was measured by fluorescence using a RiboGreen RNA quantification reagent (Invitrogen). The data were analyzed using a FluoroSkan Ascent FL microplate reader and software. RT-PCR for the FoxP3 gene was performed using 100 ng of total RNA per sample. Samples were denatured at 65°C for 10 min and incubated at 37°C for 30 min. One microliter of each cDNA was used for performing RT-PCR (Opticon, MJ Research) with SyBrGreen (Bio-Rad)). The primer sequences for the FoxP3 gene are as follows: forward 5′-CTA CGC CAC GCT CAT CCG CTG G-3′ and reverse 5′-GTA GGG TTG GAA CAC CTG CTG GG-3′ and for GAPDH as the housekeeping gene: forward 5′-ATG CAA CGG ATT TGG TCG TAT-3′ and reverse 5′- TCT CGC TCC TGG AAG ATG GTG-3′ making a 221pb amplicon.

### Statistical analysis

Continuous variables were expressed using mean ± SD and categorical variables were expressed as *N* (%) to summarize the clinical characteristics of the patients. Baseline characteristics were analyzed using a one-way ANOVA to compare asthmatic, allergic, non-asthmatic, and normal control subjects. A repeated mixed-effect model was designed with two experimental fixed factors for comparison between the three groups at baseline (first factor) and between the allergic non-asthmatic and allergic asthmatics post challenge (second factor). This second factor was analyzed as repeated measures using a compound covariance structure to appreciate the dependence among repeated measurements. The multivariate normality assumption was verified using the Shapiro–Wilk test after a Cholesky factorization, and Brown and Forsythe's variation of Levene's test was used to verify the homogeneity of variances. For some variables, values were log-transformed to stabilize variances. The reported *P*-values are based on these transformations. The results were considered significant with *P*-values ≤ 0.05. All analyses were conducted using the statistical package SAS (SAS Institute Inc., Cary, NC, USA).

## Results

### Subject characteristics

The baseline characteristics of the subjects are presented in Table 1. All allergic subjects participated in the allergen challenges and none of them experienced any allergic or asthmatic symptoms during the challenge days. No significant change in FEV_1_ was observed throughout the study period (less than 5% within and between visits in both groups).

### Treg cells but not their Foxp3 expression remain unchanged before and after allergen exposure

**Figure 1:**
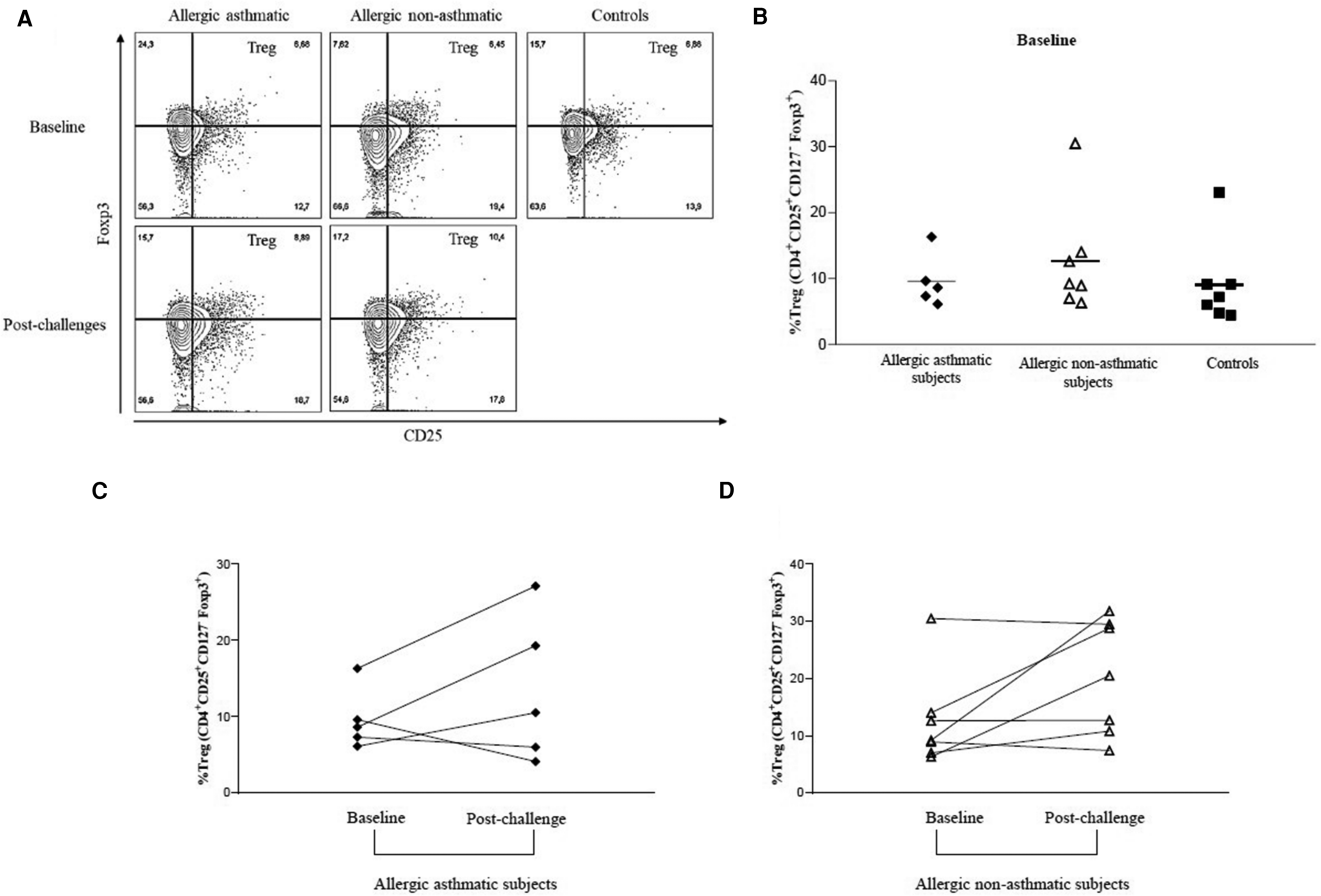
PBMC percentage of Tregs (CD4^+^CD25^+^FoxP3^+^CD127^−^) before and after allergen challenges. (**A**,**B**) Tregs were isolated from patients’ PBMC, gated, and quantified by flow cytometry using CD4^+^CD25^+^Foxp3^+^CD127^−^ discriminating markers. (**C**,**D**) Treg PBMC frequency before and after allergen challenges. Tregs from healthy controls (black square, *n* = 7), allergic non-asthmatics (white triangle, *n* = 7), and allergic asthmatics (black lozenge, *n* = 5) are expressed in terms of percentage among CD4^+^ T cells. Data are expressed using mean ± SD.

**Figure 2:**
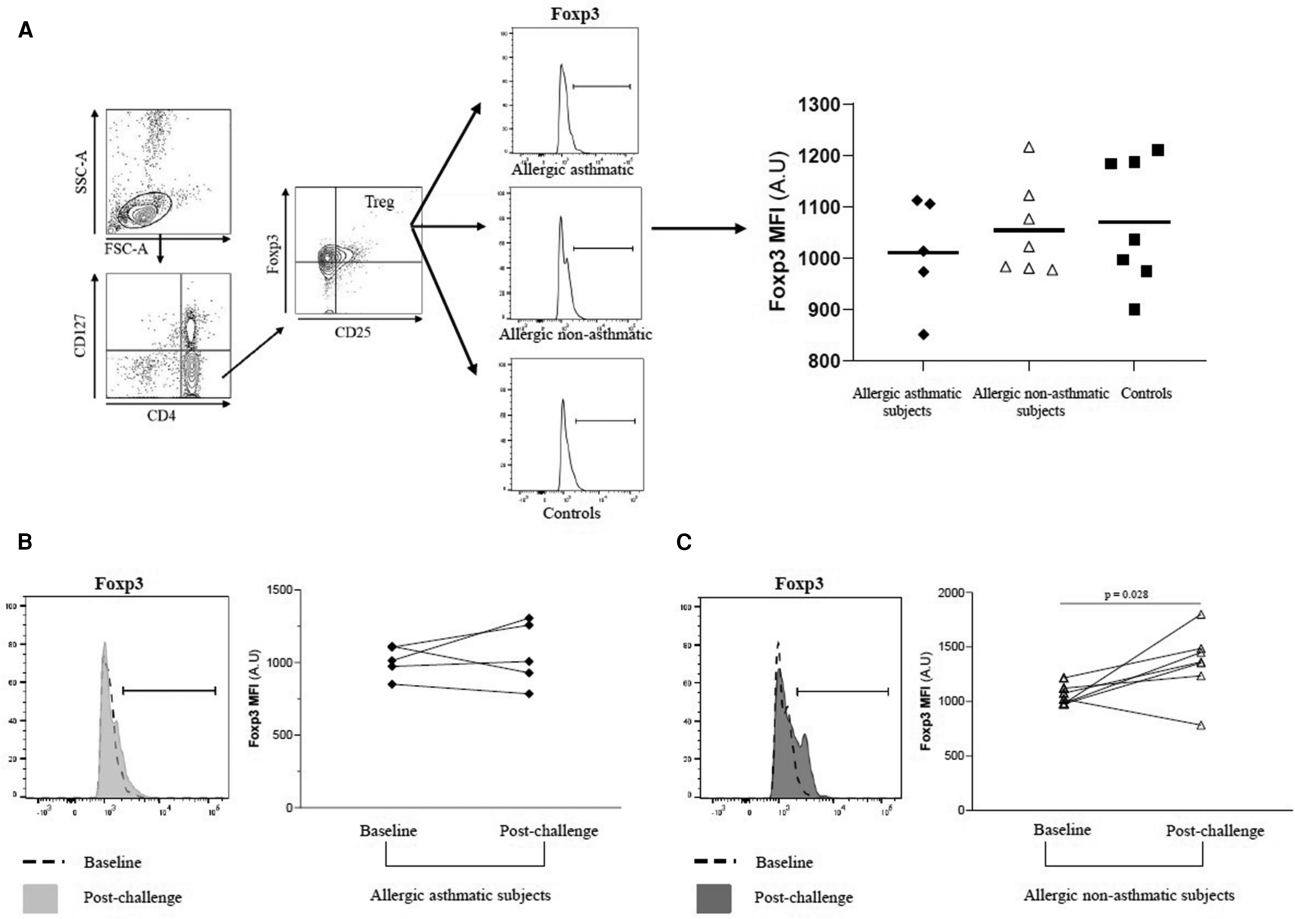
PBMC Foxp3 expression among Tregs before and after allergen challenges. (**A**–**C**) Foxp3 MFI among CD4^+^CD25^+^Foxp3^+^CD127^−^ Tregs were quantified by flow cytometry before and after allergen challenges in healthy controls (black square, *n* = 7), allergic non-asthmatics (white triangle, *n* = 7), and allergic asthmatics (black lozenge, *n* = 5). Data were expressed using mean ± SD.

First, we aimed to measure Tregs in PBMC among controls, allergic non-asthmatic, and allergic asthmatic participants at baseline. There was no significant difference in the percentage of CD4^+^CD25^+^FoxP3^+^CD127^−^ among controls, allergic non-asthmatic subjects, and allergic asthmatic subjects (7.5% ± 1%; 10.7% ± 0.1%; and 8.8% ± 0.8%, respectively) at baseline (Figure 1A). Although there was no significant change, we observed an increasing trend in the percentage of these cells in allergic non-asthmatic and asthmatic subjects after low-dose allergen challenges (Figures 1B,C). Next, we assessed Foxp3 expression by measuring its mean fluorescence intensity (MFI) among Tregs, which partly reflects their functional phenotype. At baseline, no differences in Foxp3 expression were observed among the three groups (Figure 2A). Interestingly, while no changes in Foxp3 expression in Tregs were observed in allergic asthmatic post challenge (Figure 2B), we noticed that Foxp3 expression in Tregs increased after challenges in allergic non-asthmatic subjects (*p* = 0.036) (Figure 2C). These results suggest that Tregs from both asthmatic and allergic non-asthmatics can be generated after low allergen exposure, but Tregs from allergic non-asthmatics are more potent to increase their Foxp3 expression post challenge and could be more functional.

### Tregs from allergic non-asthmatics are more suppressive than those from allergic asthmatics

**Figure 3:**
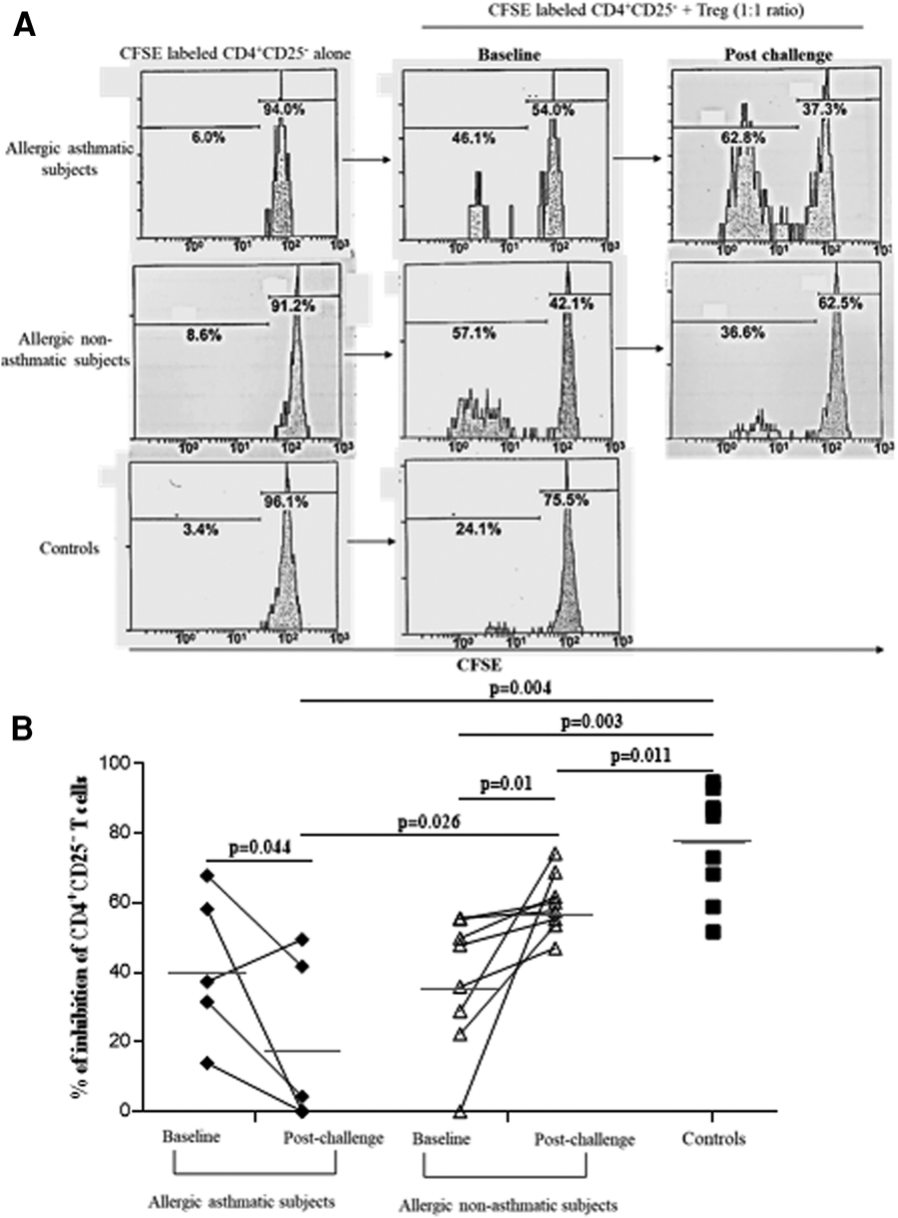
Allergic non-asthmatic Tregs are more suppressive. (**A**) Isolated Tregs (CD25^+^ FoxP3^+^CD127^−^) co-cultured with CFSE-labeled CD4^+^CD25^−^ cells from PBMC before and after allergen exposure. (**B**) Suppressive function of Tregs from healthy controls (black square, *n* = 7), allergic asthmatic subjects (black lozenge, *n* = 5), and allergic non-asthmatic subjects (white triangle, *n* = 7) are presented as median and percentage of inhibition before and after allergen exposure. Data were expressed using mean ± SD.

**Figure 4:**
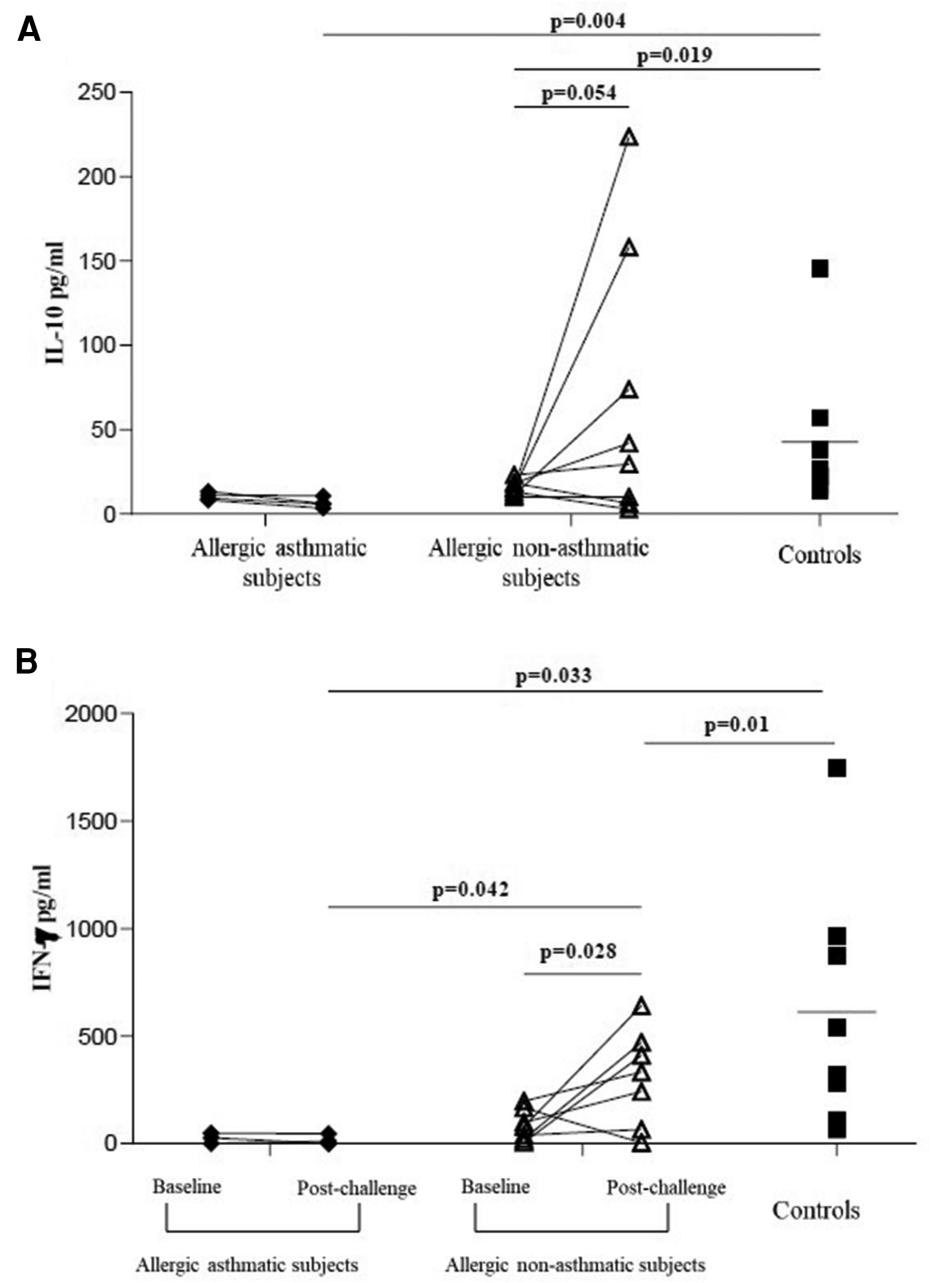
IL-10 and IFN-γ increases in culture supernatants from suppression assay. (**A**,**B**) IL-10 and IFN-γ from healthy controls (black square, *n* = 7), allergic asthmatic subjects (black lozenge, *n* = 5), and allergic non-asthmatic subjects (white triangle, *n* = 7) before and after allergen exposure were measured in supernatants from suppression assay by ELISA. Data were expressed using mean ± SD.

Following our previous results, we investigated the Treg function. We measured the suppressive function of Tregs by coculturing CD4^+^CD25^+^FoxP3^+^CD127^−^ cells with CD4^+^CD25^−^ as effector T cells. At baseline, the capacity of Tregs to suppress the proliferation of CD4^+^CD25^−^ was higher in healthy controls (76.7% ± 4.7% of inhibition) than in asthmatic (41.4% ± 7.0%; *p* = 0.004) or allergic non-asthmatic subjects (37.0% ± 6.8%; *p* = 0.003). Tregs isolated from the blood of asthmatic subjects had a lower suppressive function (41.4% ± 7.0% vs. 18.7% ± 11.5%; *p* = 0.044) than Tregs isolated from allergic non-asthmatic subjects, who had a higher suppressive function after allergen challenges (37.0% ± 6.8% vs. 59.8% ± 3.1%; *p* = 0.01) but did not reach the baseline level of healthy controls (59.8% ± 3.1% vs. 76.7% ± 4.7%; *p* = 0.011, Figures 3A,B). These results show that allergic non-asthmatic Tregs are more efficient in suppressing effector T cell proliferation after allergen exposure, while allergic asthmatic Tregs lose their suppressive function. IL-10 and IFN-γ cytokines have been shown to inhibit effector T cell proliferation in allergic diseases, and both can be produced by Tregs (18–20) Consequently, IL-10 and IFN-γ levels were measured in culture supernatants using a suppression assay. At baseline, we observed that the IL-10 level was higher in control subjects than in allergic non-asthmatic and allergic asthmatic subjects (42 ± 15 vs. 15 ± 1.5 and 10 ± 1.3 pg/ml, respectively; *p* = 0.05 and *p* = 0.004; Figures 4A,B). After allergen challenge, IL-10 production in culture supernatants tended to increase in allergic non-asthmatic subjects (15 ± 1.5 vs. 68 ± 28; *p* = 0.054; Figure 4A), but no changes were observed in allergic asthmatic subjects (10 ± 1.3 vs. 6.5 ± 1.5 pg/ml; *p* = 0.405; Figure 3B). IFN-γ followed the same production profile as that of IL-10 before and after allergen challenge. At baseline, the IFN-γ level was higher in control non-allergic subjects than in allergic non-asthmatic and allergic asthmatic subjects (450 ± 126 vs. 87 ± 23 and 19 ± 12 pg/ml, respectively; *p* = 0.0003 and *p* < 0.0001; Figure 4B). After allergen challenge, the production of IFN-γ in the culture supernatants increased significantly in allergic non-asthmatic subjects (87 ± 23 vs. 310 ± 84; *p* = 0.002; Figure 4B), while no changes were observed in allergic asthmatic subjects (19 ± 12 pg/ml at baseline vs. 15 ± 11 pg/ml post challenge). These results show that the increased suppressive function observed in allergic non-asthmatic subjects is associated with the increase in effector T cells inhibiting cytokines.

### Foxp3 gene expression increase in allergic non-asthmatic sputum

**Figure 5:**
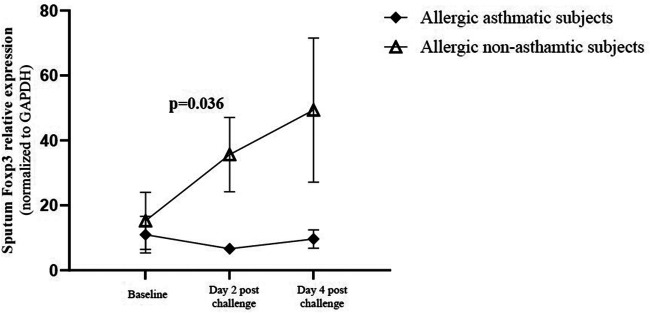
Foxp3 gene expression in induced sputum. Cells were isolated from the mucus plug and were counted using a viability marker. Live cell mRNAs were extracted and Foxp3 gene expression was quantified by qRT-PCR before and after allergen exposure. Foxp3 gene expression in healthy controls (black square, *n* = 7), allergic non-asthmatic subjects (white triangle, *n* = 7), and allergic asthmatic subjects (black lozenge, *n* = 5) was normalized on the GAPDH housekeeping gene. Data were expressed using mean ± SD.

We evaluated Foxp3 gene expression in induced sputum at baseline, 2 and 4 days after allergen challenges. Baseline Foxp3 gene expression was similar in allergic non-asthmatic and allergic asthmatic subjects. Foxp3 gene increased significantly 2 days after allergen challenges (*p* = 0.04) in allergic non-asthmatic subjects and remained elevated at day 4 post challenge. However, no change was observed in allergic asthmatic subjects over time (Figure 5).

### Anti-inflammatory cytokine and Ig measurement

**Figure 6:**
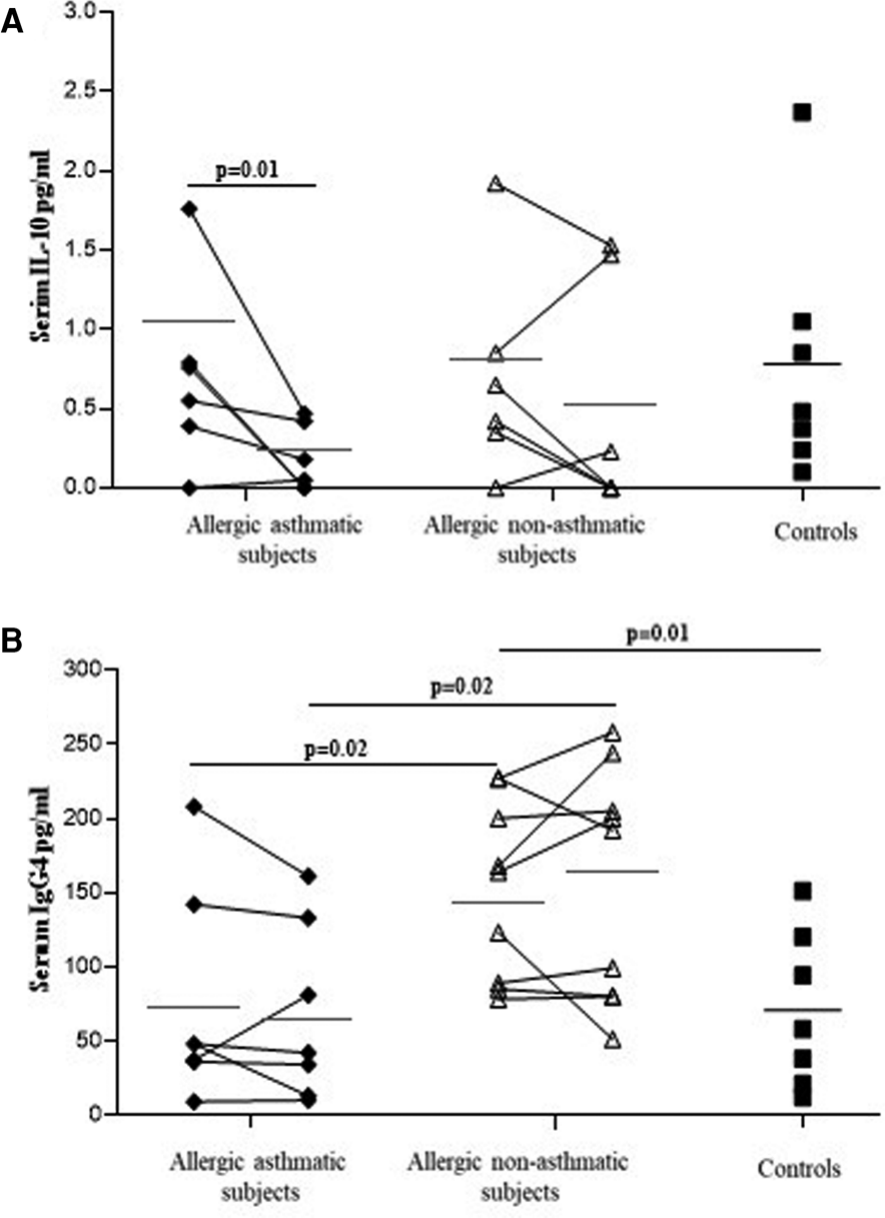
IL-10 level (**A**) decreases in allergic asthmatic patients, while total IgG4 (**B**) increases in allergic non-asthmatic patients' serum after allergen exposure. IL-10 and total IgG4 were measured in the serum of healthy controls (black square, *n* = 7), allergic asthmatics (black lozenge, *n* = 6–7) and allergic non-asthmatic subjects (white triangle, *n* = 7) by ELISA before and after allergen exposure. Data were expressed using mean ± SD.

IL-10, TGF-β, IgA, and total IgG4 levels were measured using ELISA in serum. No differences were observed in IL-10 among healthy controls, allergic asthmatics, and allergic non-asthmatics at baseline. In asthmatic subjects, IL-10 showed a significant decrease after allergen challenges, while no significant change was observed in allergic non-asthmatic subjects (Figure 6A). Total IgG4 was higher in allergic non-asthmatic subjects than in healthy controls and asthmatic subjects (164 ± 22 vs. 77 ± 21, *p* = 0.01; and 76 ± 30 µg/ml, *p* = 0.02, respectively). No change was observed between baseline and post-challenge conditions in allergic non-asthmatic and allergic asthmatic subjects (Figure 6B). No significant difference was found in serum among the three groups for TGF-β at baseline or after allergen challenge (Supplementary Figure S3). The levels of IgA were similar at baseline among the three groups and after allergen challenge in the asthmatic and allergic non-asthmatic groups (Supplementary Figure S4).

## Discussion

The present work demonstrates that the function of regulatory T cells is decreased in allergic non-asthmatic and allergic asthmatic subjects compared with healthy controls. Repeated exposure to low doses of allergen restored the suppressive function of Tregs in allergic non-asthmatic subjects, while it decreased it in allergic asthmatic subjects. This restored suppressive function is associated with increased Foxp3 frequency in Tregs and is correlated with increased Foxp3 gene expression in cells from induced sputum after allergen challenge in allergic non-asthmatics.

T regulatory cell function and number have been studied in allergic disease, but there are discrepancies among studies. Most studies showed a decrease in the number and the function of Tregs in allergic diseases (21–24). However, Lee et al. showed an increase in Tregs and Foxp3 expression in the blood of children with severe asthma or persistent allergic rhinitis compared with milder disease (25). This could be explained by the fact that patients with severe asthma or allergic rhinitis were treated with corticosteroids, which have been shown to improve Treg function (26). Tregs and their suppressive activity have been shown to be decreased in the blood depending on the dose and type of allergen (27). Tregs from grass-pollen-allergic donors failed to inhibit proliferation of effector T cells at high allergen concentrations, while Tregs from non-atopic donors retained their regulatory capacity even at these high concentrations. Foxp3 protein expression within Tregs was shown to be significantly decreased in the blood of asthmatic patients (22). Foxp3 expression was higher in bronchoalveolar lavage (BAL) after allergen challenge (4). Despite this increase of Foxp3 after allergen challenge, the levels of Th2 cytokines are still high in BAL, suggesting an impaired function of T regulatory cells in asthmatic subjects. Foxp3 is well described as a transcription factor that confers T cells their regulatory phenotype and function (28–30). In this study, we found no difference in the baseline percentage of Tregs or Foxp3 among Tregs among the three studied groups. However, the Treg function was decreased in allergic subjects with and without asthma when compared with non-allergic non-asthmatic individuals. Interestingly, in allergic non-asthmatic individuals, repeated exposure to low doses of allergen increased the suppressive activity of Tregs and their Foxp3 expression. In addition, Foxp3 gene expression increased in induced sputum in allergic non-asthmatic subjects, whereas in asthmatics, the suppressive function of Tregs was significantly decreased, and no change was observed in Foxp3 in Tregs and in induced sputum, suggesting that Foxp3 regulation in allergic non-asthmatics differs from that in allergic asthmatics. Regulatory T cells are heterogeneous in phenotype and their manner of generation. They can be classified into two major categories: thymus-derived natural Treg cells and those induced in the periphery under tolerogenic conditions (31). Both naturally occurring thymus-derived regulatory T cells and inducible regulatory T cells suppress the development of allergic diseases mainly mediated by the suppressive cytokines IL-10 and TGF-β (32), and other studies have demonstrated that Tregs can also produce IFN-γ (19, 33, 34). Inducible Tregs producing IL-10 are reduced in the blood of unstable severe asthmatic patients compared with patients with mild and severe stable asthma (35). Although the detection level was low, we observed a decrease in IL-10 levels in the serum of allergic asthmatic subjects post-allergen challenges, whereas no significant change was found in the non-asthmatic group. IL-10, TGF-β, and to a lesser extent, IFN-γ can counteract effector T cells by inhibiting their polarization and proliferation (18, 36). We observed an increase in IL-10 and IFN-γ levels in our coculture medium, which correlated with greater inhibition of effector T cell proliferation by Tregs in allergic non-asthmatic subjects post challenge. In contrast, the lack of increased IL-10 or IFN-γ production correlated with a reduced suppressive Treg function in allergic asthmatic subjects post challenge and may contribute to an increased Th2 response in these subjects.

In allergic diseases, IgG4 counteracts immediate hypersensitivity symptoms through different blocking mechanisms such as allergen trapping, competes with IgE to bind CD23, and so on (37). We observed that the total IgG4 level was increased in allergic non-asthmatics, whereas no change was observed in the level of IgA. A study by Meiler et al. showed that allergen-specific Tregs induce IgG4 but have no effect on IgA (38). Total IgG4 can also be induced by naturally occurring Tregs (CD4^+^CD25^+^CD127^low^Foxp3^+^) via GITR/GITR-L interaction and IL-10 (39), suggesting that an increased number of Tregs and increased IL-10 levels observed in allergic non-asthmatics post challenge may explain the rise in the total IgG4 level. However, in this study, we measured only total IgG4 and, therefore, we could not correctly interpret IgG4 data without performing an allergen-specific IgG4 measurement. Using this model of repeated low-dose allergen challenge, we found that eosinophil counts were still high in the lower airways of allergic asthmatics but not in non-asthmatic allergic subjects after 4 days of daily low-dose challenges (40). This may be attributed to the development of effective immune regulation in allergic non-asthmatics. Altogether, these data suggest that Treg phenotype and functions are partially maintained in allergic non-asthmatic subjects, whereas they are completely lost in allergic asthmatics upon low-dose allergen exposure. This study has some limitations. First, the number of subjects is low, and some included subjects could not produce enough sputum during the protocol, resulting in low sample availability compared with the initially recruited subjects. Second, we focused on Treg phenotype and functions to compare our groups, but we did not investigate allergen-specific Treg (Tr1). Tr1 is induced following chronic allergen exposure and does not express Foxp3 but produces IL-10, TGF-β, and cell contact-inhibiting molecules in high quantities (41). Further experiments should be performed to better identify these Treg subsets. Other cells actively participate in immune regulation during allergic diseases, such as dendritic cells, regulatory B cells (Bregs) (42, 43), and the recently discovered CD8^+^ regulatory cells (CD8^+^CD25^+^Foxp3^+^) (44, 45), and we will study these cells in our future work. Third, our study was designed only for short-term exposure to allergen, but it would be interesting to investigate whether the suppressive function of allergic non-asthmatic subjects would be maintained upon long-term exposure to low-dose allergen. In this study, three out of five asthmatic patients and four out of seven allergic non-asthmatic patients were challenged with allergens considered to be perennial (house dust mites and cat hair); consequently, they may have been continuously exposed to these allergens and they may have different immunological parameters compared to patients exposed to seasonal allergen regarding the baseline state of our study. Baseline analysis comparing asthmatic allergic and non-asthmatic allergic subjects did not reveal any differences between the use of seasonal or perennial allergens for each group. However, the limited number of subjects in this study could mask the difference between perennial and seasonal challenges, as perennial allergen challenges are known to induce markedly different and distinct symptoms (40, 46) and, therefore, should be taken into consideration in further studies.

Nonetheless, the discrepancy in the suppressive Treg short-term responding profile in allergic non-asthmatic and allergic asthmatic individuals may be relevant, requiring further studies to unravel the mechanisms of impaired Treg function between allergic diseases.

In conclusion, our study suggests that, unlike allergic asthmatic subjects, allergic non-asthmatic subjects were able to develop short-term mechanisms of tolerance when they were exposed to repeated allergen challenges.

## Data Availability

The original contributions presented in the study are included in the article/**Supplementary Material**, further inquiries can be directed to the corresponding author.
