# Supplementary material for: Discrepancy in the suppressive function of regulatory T cells in allergic asthmatic vs. allergic rhinitis subjects upon low-dose allergen challenges
Source: Front Allergy. 2023 Dec 1;4:1296601. doi: 10.3389/falgy.2023.1296601 (PMC10722309; doi:10.3389/falgy.2023.1296601)
Supplement: Supplementary file 1 [file Datasheet1.pdf]

Figure S1

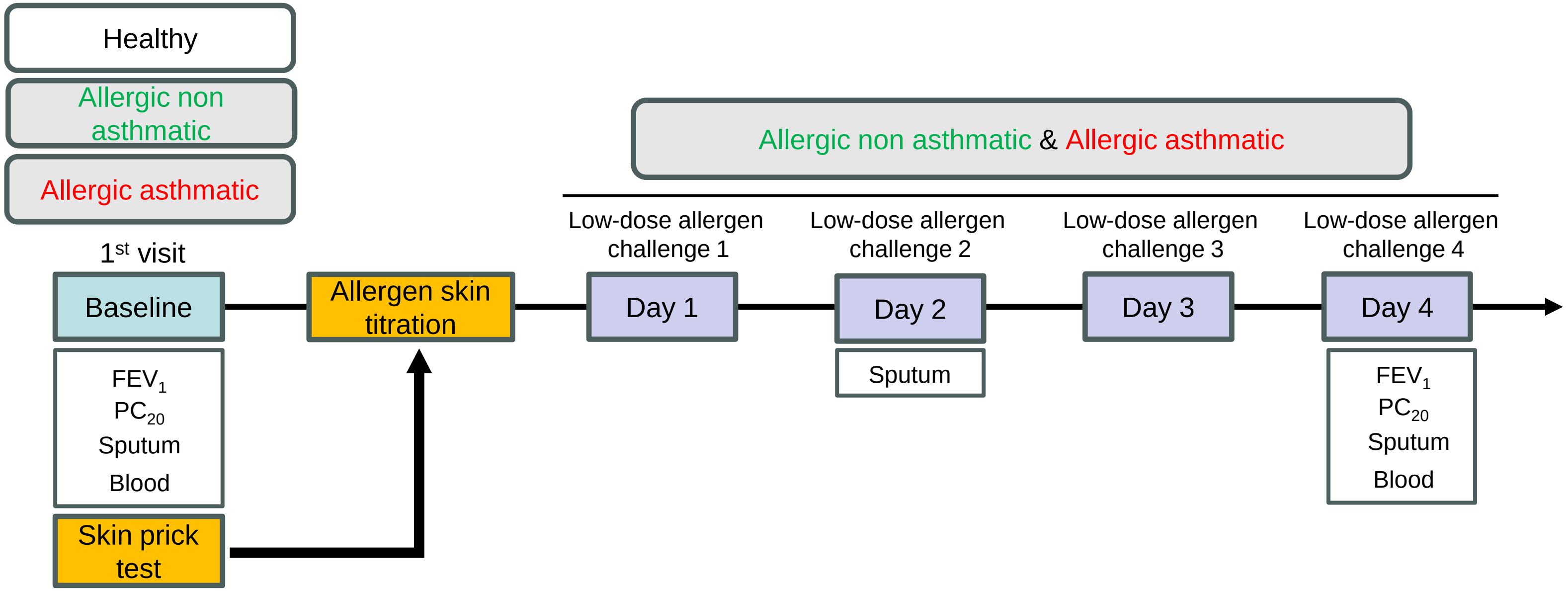

Healthy
Allergic non asthmatic
Allergic asthmatic
1st visit
Baseline
FEV1
PC20
Sputum
Blood
Skin prick test
Allergen skin titration
Allergic non asthmatic & Allergic asthmatic
Low-dose allergen challenge 1
Low-dose allergen challenge 2
Low-dose allergen challenge 3
Low-dose allergen challenge 4
Day 1
Day 2
Sputum
Day 3
Day 4
FEV1
PC20
Sputum
Blood

Figure S2

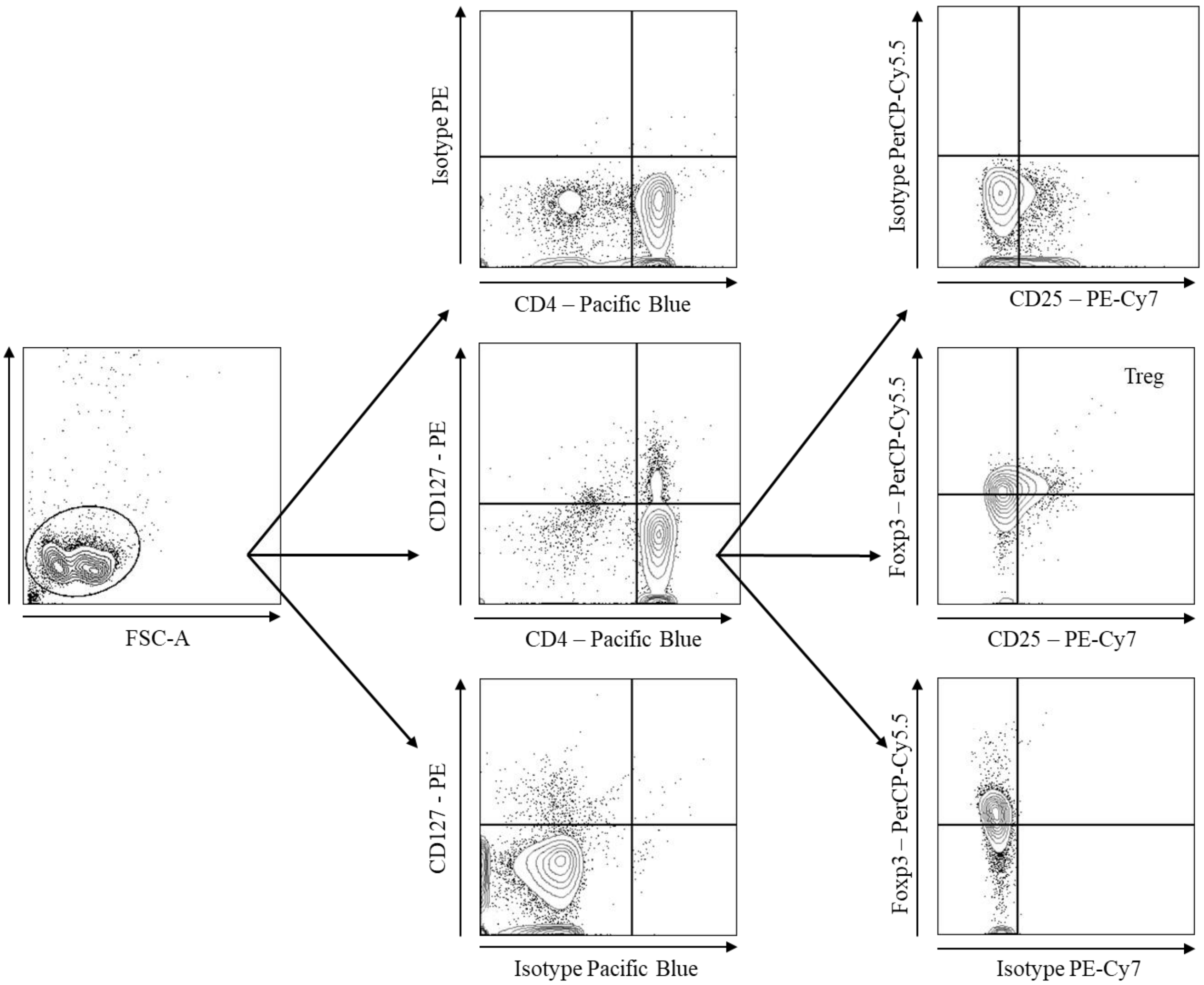

Figure S2: Gating strategy after immunostaining to determine regulatory T cells (Tregs) subset.

Figure S3

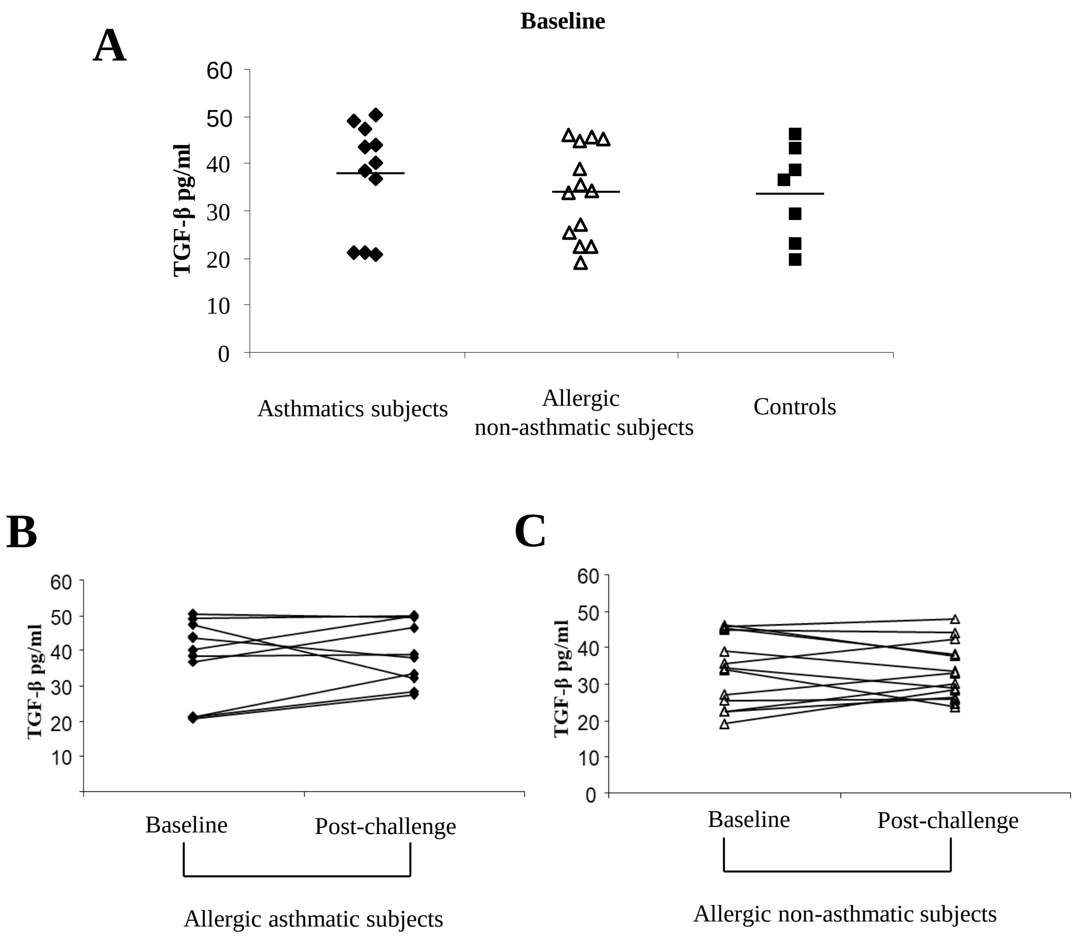

Figure S3: Serum TGF-β before and after allergen exposure. TGF-β levels were dosed in serum of healthy controls (black square), allergic asthmatics (black lozenge) and allergic non-asthmatic subjects (white triangle) by ELISA before and after allergen exposure. . Data were expressed using mean ± SD.

Figure S4

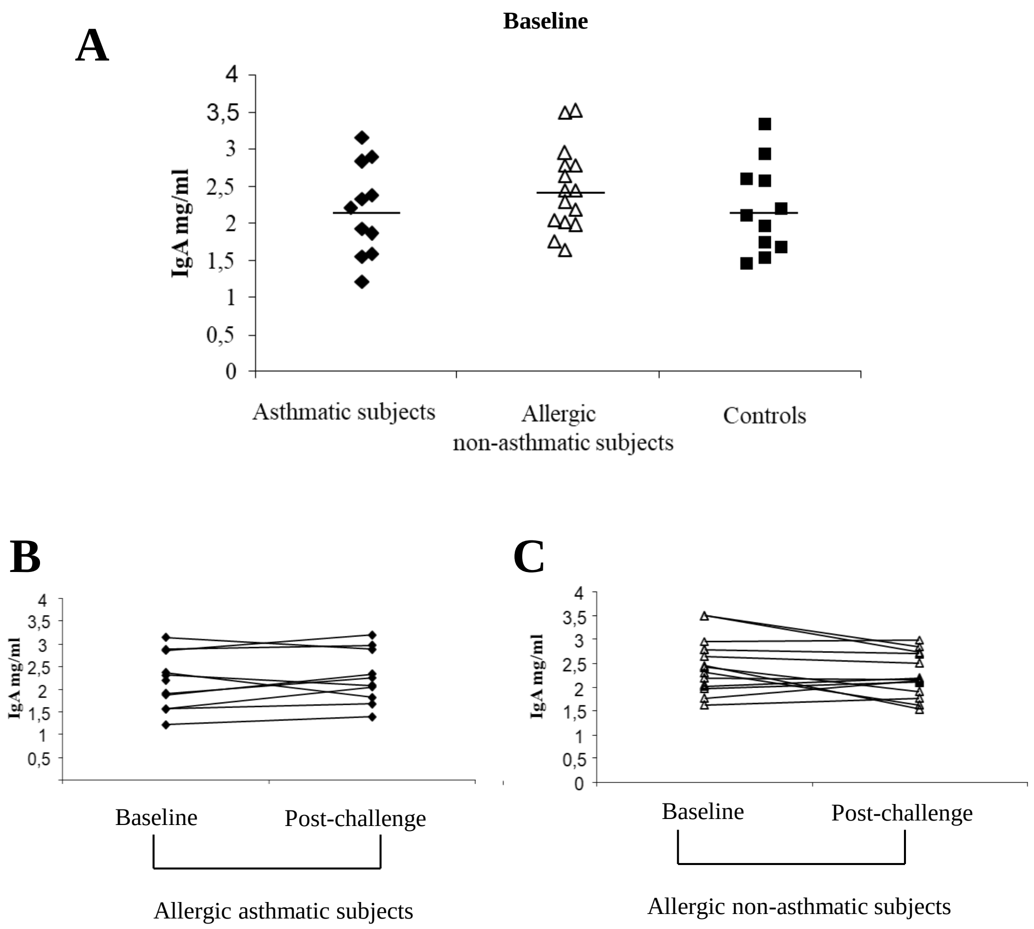

Figure S4: Serum IgA before and after allergen exposure. IgA levels were dosed in serum of healthy controls (black square), allergic asthmatics (black lozenge) and allergic non-asthmatic subjects (white triangle) by ELISA before and after allergen exposure. . Data were expressed using mean ± SD.
