# Supplementary material for: Discrepancy in the suppressive function of regulatory T cells in allergic asthmatic vs. allergic rhinitis subjects upon low-dose allergen challenges
Source: Front Allergy. 2023 Dec 1;4:1296601. doi: 10.3389/falgy.2023.1296601 (PMC10722309; doi:10.3389/falgy.2023.1296601)
Supplement: Supplementary file 2 [file Datasheet2.pdf]

Table S1: Skin prick tests and allergen-sepecific challenges of allergic subjects

	Positive prick test results	Major allergen sensitivity (based on positive skin prick test)	Allergen-specific challenges	Data available for each test during the study
**Asthmatic allergic subjects**				
A01	cat, dog and horse hair, ragweed, grass, ryegrass, house dust	cat hair	cat hair	No sputum
A02	D.Farinae, D.Pteronyssinus, cat and horse hair, feather, house dust, ryegrass, grass, ragweed,	D.Farinae/D.Pteronyssinus	D.Pteronyssinus	No sputum and blood at day 4
A03	birch pollen	birch pollen	birch pollen	No sputum
A04	D.Farinae, D.Pteronyssinus	D.Pteronyssinus	D.Pteronyssinus	No sputum
A05	cat and horse hair, house dust	cat hair	cat hair	No blood at day 4
A06	D.Farinae, D.Pteronyssinus, cat, dog and horse hair, birch pollen, oak, mugwort, house dust, ryegrass, grass, ragweed,	D.Pteronyssinus	D.Pteronyssinus	Yes
A07	cat and dog hair, ragweed, house dust	cat hair	cat hair	Yes
A08	grass, birch pollen, ragweed, cat hair, ryegrass, D.Farinae, oak, mugwort	grass	grass	Yes
A09	cat, dog and horse hair, ragweed, grass, ryegrass, house dust, feather	cat hair	cat hair	Yes
A10	dog and horse hair, oak, grass, ragweed, ryegrass, house dust, Aspergillus fumigatus	grass	grass	Yes
A11	D.Pteronyssinus, D.Farinae, cat and horse hair, grass, ragweed, mugwort, house dust, feather	D.Pteronyssinus	D.Pteronyssinus	No blood at day 4
**Allergic non-asthmatic subject**				
R01	cat hair, birch pollen	birch pollen	birch pollen	No sputum
R02	cat hair, mugwort, grass	cat hair	cat hair	Yes
R03	D.Farinae, D.Pteronyssinus, house dust	D.Farinae	D.Farinae	No sputum
R05	cat hair, grass, ryegrass	cat hair	cat hair	No sputum and no blood at day 4
R06	cat hair, birch, oak, ryegrass, grass, ragweed, mugwort	grass	grass	No blood at day 4
R07	cat hair, birch, house dust, mugwort	cat hair	cat hair	Yes
R09	cat hair, birch, mugwort, grass	grass	grass	Yes
R10	oak, birch, grass, mugwort, ragweed, D.Farinae, D.Pteronyssinus	grass	grass	Yes
R11	D.Farinae, D.Pteronyssinus, house dust	D.Pteronyssinus	D.Pteronyssinus	Yes
R12	cat and dog hair, ragweed, house dust, ragweed, mugwort, grass, oak, birch, Aspergillus fumigatus, D. Farinae, D.Pteronyssinus, feather	grass	grass	Yes
R13	D.Pteronyssinus ,D. Farinae, grass, ragweed	D.Pteronyssinus	D.Pteronyssinus	Yes
R14	grass, birch, mugwort	grass	grass	No sputum

Table S2: Skin prick tests wheal for each allergen and subjects

	Positive prick test results													
**Allergen**	Cat hair	Dog hair	Asp. fumigatus	Ragweed	Grass	Ryegrass	House dust	D. Farinae	D. Pteronyssinus	Feather	Birch pollen	Oak	Mugwort	Horse hair
**Asthmatic allergic subjects**	**Skin wheal (mm)**													
A01	**<u>5</u>**	4	0	3	4	0	4	1	1	0	0	0	0	5
A02	4	0	0	5	5	5	4	6	**<u>6</u>**	4	0	0	0	5
A03	0	0	0	0	0	0	0	0	0	0	**<u>4</u>**	0	0	0
A04	0	0	0	0	0	0	0	4	**<u>8</u>**	1	0	0	0	0
A05	**<u>4</u>**	2	0	1	0	1	3	0	2	0	1	1	1	3
A06	4	2	0	3	7	5	3	5	**<u>8</u>**	2	4	3	4	5
A07	**<u>6</u>**	3	0	5	0	0	4	2	0	0	2	0	0	2
A08	3	1	0	6	**<u>8</u>**	3	2	3	0	1	5	7	5	1
A09	**<u>8</u>**	3	0	2	5	3	4	0	0	3	0	1	2	5
A10	3	0	3	2	**<u>12</u>**	7	4	3	3	0	3	5	3	6
A11	4	0	0	3	5	4	3	4	**<u>8</u>**	3	0	1	2	6
**Allergic non-asthmatic subject**	**Skin wheal (mm)**													
R01	5	0	0	0	0	0	0	0	0	0	**<u>6</u>**	2	0	0
R02	**<u>4</u>**	1	0	0	3	2	1	2	1	0	0	0	4	0
R03	0	0	0	0	2	0	3	**<u>9</u>**	6	2	0	0	0	0
R05	**<u>4</u>**	2	0	0	4	4	2	0	0	0	0	0	1	2
R06	3	0	0	5	**<u>9</u>**	5	2	2	0	0	6	8	5	0
R07	**<u>5</u>**	2	0	0	0	0	3	1	2	0	4	2	3	2
R09	3	0	0	4	**<u>7</u>**	2	0	0	2	0	3	0	2	1
R10	0	0	0	3	**<u>10</u>**	6	0	7	8	2	6	5	7	0
R11	0	0	1	0	0	0	3	9	**<u>10</u>**	2	0	0	0	0
R12	5	4	3	4	**<u>11</u>**	4	5	7	10	4	7	6	6	8
R13	4	0	0	4	7	0	0	6	**<u>9</u>**	0	0	0	0	0
R14	0	0	0	1	**<u>8</u>**	2	0	0	0	0	0	0	3	0

Legend: Bold and underlined skin wheal measurements correspond to the allergen used for challenges.
